# The burden of obesity in the current world and the new treatments available: focus on liraglutide 3.0 mg

**DOI:** 10.1186/s13098-017-0242-0

**Published:** 2017-05-31

**Authors:** Marcio C. Mancini, Maria Edna de Melo

**Affiliations:** 1 Obesity and Metabolic Syndrome Unit, Endocrinology and Metabolism Service, Clinics Hospital, São Paulo University Medical School, São Paulo, Brazil; 2 Laboratory of Carbohydrates and Radioimmunoassay LIM-18, São Paulo University Medical School, São Paulo, Brazil; 3 Endocrinology and Metabolism Service Secretariat, Av. Dr. Enéas de Carvalho Aguiar, 255, 7º andar, sala 7037, São Paulo, SP 05403-000 Brazil

**Keywords:** Obesity, Anti-obesity drugs, Appetite regulation, Weight loss, Liraglutide

## Abstract

The prevalence of obesity increases worldwide. Treating obesity and its associated health problems has a significant economic impact on health care systems. The unsatisfactory long-term outcomes observed in the obesity treatment are due to its complex pathophysiology and the inherent difficulties associated with maintenance of lifestyle modifications. Determined by genetic and environmental factors, obesity has been officially recognized as a chronic disease, an action that allowed the recognition of anti-obesity drugs as legitimate therapeutic options to address the growing obesity endemic. Like other chronic diseases, obesity requires long-term treatment. Pharmacological interventions, when used as an adjunct to lifestyle changes, are useful to facilitate clinically meaningful weight loss, which may impact on obesity-associated comorbid conditions. In the past, medications for weight reduction were limited. However, the landscape has changed and new drugs provide additional options for weight management. Among the new drugs, liraglutide is the most studied, especially regarding its effects on the limbic system. As an adjunct to a reduced-calorie diet and increased physical activity, treatment with liraglutide 3.0 mg provides a statistically significant and clinically meaningful weight loss. Liraglutide is a glucagon-like peptide 1 (GLP-1) receptor agonist that shares 97% homology to native GLP-1. Receptor agonists of GLP-1, including liraglutide, have emerged as effective therapies for type 2 diabetes and obesity. This review will address the major findings concerning the central regulation of appetite and the main studies that evaluated new drugs for obesity treatment, with a greater focus on liraglutide 3.0 mg.

## Background

More than just a consequence of poor lifestyle choices, obesity is a disease that is complex, polygenic, multifactorial, chronic and resistant to many forms of treatment. Obesity predisposes to the development of cardiovascular diseases (CVD), type 2 diabetes mellitus (T2D), hypertension and numerous other conditions [1].

During the period between 1980 and 2014, the world prevalence of obesity more than doubled. According to the World Health Organization (WHO) more than 1.9 billion adults over 18 years of age were overweight (38% of men and 40% of women), of these over 600 million were obese (11% of men and 15% of women) in 2014. Forty-two million children under 5 years of age were overweight or obese in 2013. In emerging countries, the increase of childhood overweight and obesity has been more than 30% higher than that of developed countries [2]. For instance, in Brazil, 16.8% of men and 24.4% of women were obese, while 56% of the adult population were overweight in 2013 [3].

The body mass index (BMI) is an attempt to quantify the amount of tissue mass (muscle, fat, and bone) individually and BMI categories are defined as follows: lean below 18.5, normal weight 18.5–24.9, overweight 25–29.9, and obese over 30 kg/m^2^ [2]. In accordance with WHO, normal weight East Asians have a BMI 18.5–23, overweight 23–27.4 and obese over 27.5 kg/m^2^ [4]. BMI has significant limitations since it does not always exhibit the risk of other chronic weight-related conditions [5]. Sharma et al. have proposed a simple clinical and functional staging system, namely Edmonton Obesity Staging System (EOSS). When used together with other anthropometric classification, EOSS provides a more accurate measurement of obesity-related health risks, because it incorporates the presence of comorbidities to aid decision-making in clinical practice [6], which is important, given that metabolically healthy obese comprise approximately 20% of obese individuals [7].

In the United States, the increased prevalence of obesity is responsible for almost $40 billion of increased medical spending through 2006, including $7 billion in Medicare prescription drug costs. Finkelstein et al. have shown the extent to which excess weight increased annual medical spending: the costs of overweight and obesity could have been as high as $78.5 billion in 1998 and $147 billion in 2008 [8]. Based on data from 2000 to 2005, Cawley and Meyerhoefer estimated that the United States medical care costs of obesity-related illness in adults is $209.7 billion, which corresponds to 20.6% of the national health expenditures [9].

Factors leading to the development of obesity have been extensively studied in recent years. The central regulation of appetite, especially with regard to the hedonic appetite, is a field highly exciting. Recently, new medications have been approved for the treatment of obesity in the United States: liraglutide, phentermine/topiramate, naltrexone/bupropion and lorcaserin.

Liraglutide is a glucagon-like peptide 1 (GLP-1) receptor agonist that shares 97% homology to native GLP-1, extending the circulating half-life of GLP-1 from 1 to 2 min to 13 h. Liraglutide was first approved for the treatment of T2D in Europe in 2009 [10]. Among the new obesity drugs, liraglutide is the most studied, especially regarding its important effects through actions on the limbic system [11].

This review will address the major findings concerning the central regulation of appetite and the main studies with the new anti-obesity drugs, with a greater focus on liraglutide 3.0 mg.

## Pathophysiology of obesity

Besides the well-known factors that lead to obesity, such as increase in energy intake by ingestion of high energy-dense processed foods and reduction in physical activity, there are several different factors to be considered. Some putative contributors to obesity include the gut microbiota, endocrine disruptors, epigenetics, increasing maternal age, greater fecundity among people with higher adiposity, assortative mating, sleep debt, pharmaceutical iatrogenesis, reduction in variability of ambient temperatures, and intrauterine and intergenerational effects [12]. Human adiposity and the predisposition towards weight gain are influenced by multiple genes, and the most probable estimate of the heritability of body fat in humans range from about 25 to 75% [13]. Gene mutations that are singly sufficient to cause human obesity are extremely rare. Almost 176 cases of human obesity due to mutations in more than ten different genes have been reported. Noticeably, almost all of these mutations are bounded in the leptin/melanocortin pathway of the hypothalamus, which is critical in the adjustment of whole-body energy homeostasis [14].

The study of common obesity or polygenic obesity is approached by linkage studies, candidate gene association studies and genome-wide association studies (GWAS), in an attempt to find associations between genetic variations and an obesity-related trait. Until now, GWAS had identified almost 100 loci associated with obesity-related traits, among them, the most relevant is the rs9939609 polymorphism located in the FTO gene [15, 16].

The inability to limit excessive food intake is probably a key process that contributes to uncontrolled weight gain, mainly because it seems to be satisfying to eat a more palatable energetic and obesogenic type of food. The high hedonic value and tempting foods are the main culprits to overeat or eat beyond the immediate metabolic need (food reward behaviour) [17]. The relationship between energy intake and expenditure is modulated not only by environmental and behavioural factors but also by genetic determinants and neuroendocrine feedback mechanisms. These mechanisms are regulated by the hypothalamus, the central site for the homeostatic regulation of body weight. The hypothalamus integrates peripheral hormonal signals from the gastrointestinal tract [ghrelin, cholecystokinin, peptide YY, pancreatic polypeptide (PP), GLP-1], pancreas (insulin) and adipose tissue (leptin), that modify central orexigenic [e.g. neuropeptide Y (NPY)], agouti-related peptide (AgRP) and anorexigenic [e.g. alpha-melanocyte stimulating hormone (α-MSH), a proopiomelanocortin (POMC)-derived peptide], cocaine- and amphetamine-regulated transcript [CART]) neuropeptides [18].

**Fig. 1:**
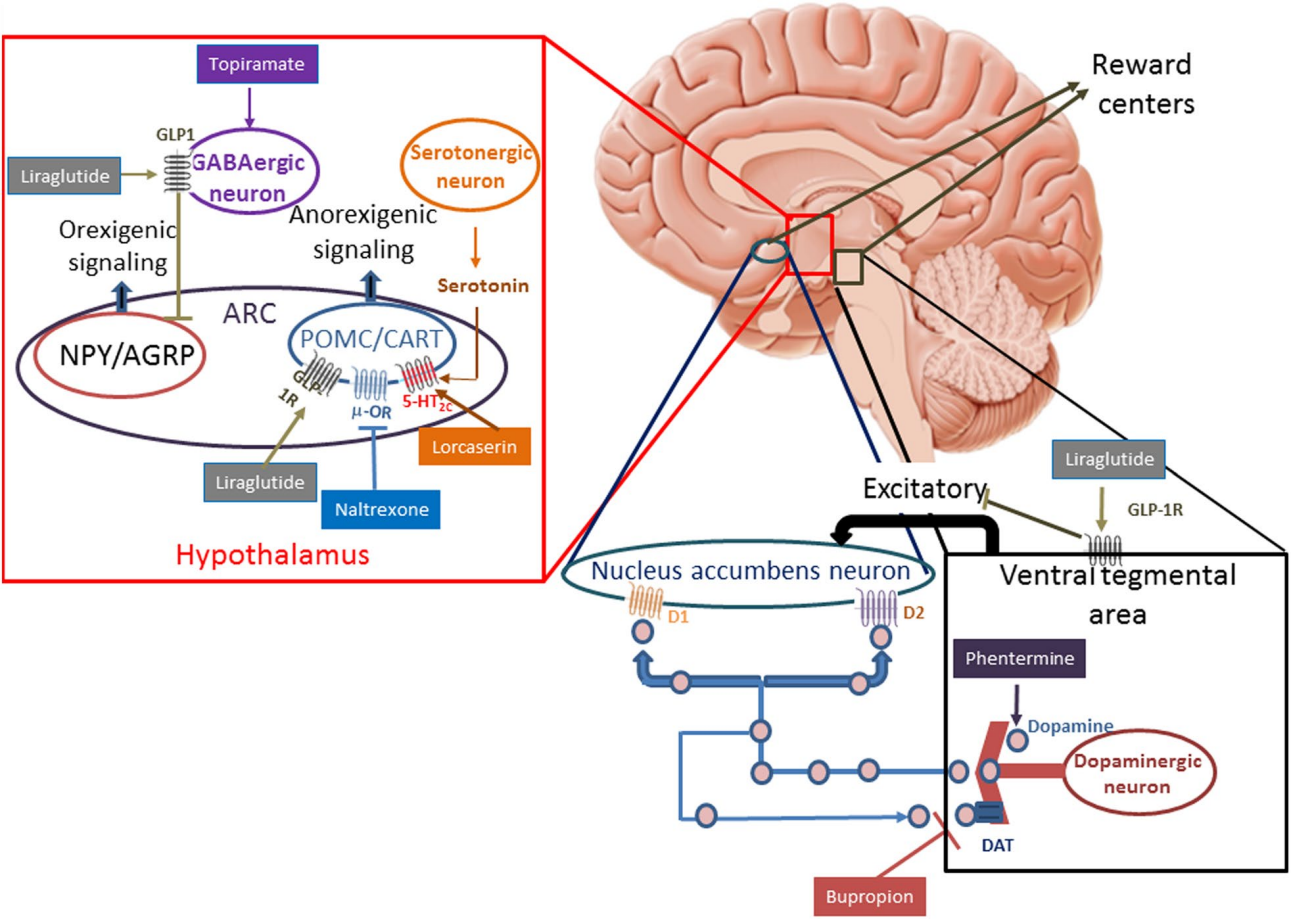
Mechanism of action of antiobesity drugs. 5-HT2C-R: 5-hydroxytryptamine (serotonin) 2C receptor; *ARC* arcuate nucleus, *CART* cocaine and amphetamine-regulated transcript, *D1* dopamine receptor D1, *D2* dopamin receptor D2, *DAT* dopamine transporter, *GABA* gamma-aminobutyric acid, *GLP-1R* glucagon-like peptide-1 recepto, *NPY/AgRP* neurons expressing neuropeptide Y and agouti- related peptide, *POMC* proopiomelanocortin, *μ-OR* μ-opioid receptor (Adapted from Kim et al., Baggio et al. and Wang et al. [19–21])

In animal studies, GLP-1 directly stimulates POMC/CART neurons and indirectly inhibits neurotransmission in neurons expressing NPY and AgRP via gamma-amino butyric acid (GABA)-dependent signalling [19–21]. These findings indicate that the GLP-1 receptors (GLP-1R) on POMC/CART-expressing arcuate (ARC) neurons likely mediate liraglutide-induced weight loss (Fig. 1). The weight loss is reduced by local blockade of GLP-1R on ARC neurons with an antagonist of GLP-1R [22]. Although well documented in animal models, the action of liraglutide in the human hypothalamus is more difficult to be demonstrated. Studies with functional magnetic resonance imaging (fMRI) in humans have not identified modifications on its neuronal activity with the use of liraglutide; this absence of signal can be due to the small size of hypothalamus and its immediacy to the sinuses [23, 24].

The hedonic appetite regulation is carried out in the limbic regions such as the hippocampus, amygdala, nucleus accumbens, ventral tegmental area, cingulate gyrus, orbitofrontal cortex, insula and prefrontal cortex. These areas are involved in the development of the conditioned response and reward. Although classical neurotransmitters involved in this network are dopamine, serotonin, and GABA, several animal studies emphasize the contribution of GLP-1 in the system [25, 26]. Hsu et al. assessed the dietary behavior of rats that received exedin-4 in the ventral portion of the hippocampal formation. In addition to reducing total energy intake and weight, it was observed a significant decrease in fat intake and an increase in the standard feed consumption, when they were allowed to choose what food to eat. This finding highlights the importance of GLP-1 in brain regions involved in the control of the learned and motivational behaviors in food consumption [26].

Farr et at. identified, by immunohistochemical analysis, the presence of GLP-1R in hypothalamic nuclei, medulla oblongata, area postrema and parietal cortex of humans brains [23]. The inferior parietal cortex is part of the attention network, which can be activated by important or highly desirable stimuli, like palatable food. Humans taking liraglutide presented in fMRI a decrease in activation of parietal cortex in response to more desirable food. This parietal activation in response to high-energy food is inversely correlated to weight loss. Patients with the lowest inferior parietal activation reported it would be less pleasant to eat while on liraglutide, when on fasting. In this same study, it was reported a decrease in activation at insula and putamen in patients taking liraglutide when exposed to palatable food cues. The insula participates in the saliency processing and saciety, in turn, putamen seems to contribute in the processing of food reward [23]. A previous study with exenatide, another GLP-1 receptor agonist (derived from exendin-4), also showed a decreased brain response to palatable food pictures in insula, amygdala, putamen, and orbitofrontal cortex [27].

Another recent study employing fMRI determined the effects of endogenous GLP-1 (using a GLP-1 antagonist) and of liraglutide on central nervous system (CNS) activation in healthy lean individuals as well as in T2D patients. Endogenous GLP-1 was shown to affect central responsiveness to palatable food consumption. In comparison to healthy lean subjects, T2D patients presented reduced activation of the right insula by chocolate milk. In obese T2D patients, liraglutide improved the observed deficit in response to palatable food, which may contribute to the weight loss observed with liraglutide [28].

An aspect that has been recently considered in the study of obesity is hormonal adaptations to weight loss. Sumithran et al. enrolled 50 overweight or obese nondiabetic patients in a 10-week weight loss program consisting of a very-low-energy diet. At the end of 10 weeks, after a mean weight loss of 13.5 ± 0.5 kg, there were significant reductions in the concentrations of the anorectic peptides leptin, peptide YY, cholecystokinin, insulin and amylin. There were also increases in the concentrations of the orexigenic peptides ghrelin, glucose-dependent insulinotropic polypeptide (GIP) and PP. One year after the initial weight loss, there were still significant differences in the mean concentrations of these peptides in comparison to baseline; GLP-1 levels were also lower than baseline. The authors concluded that the modifications in the circulating mediators of appetite that encourage weight regain persist after one year of the weight loss. They highlight the importance of strategies to counteract this change in order to prevent obesity recidivism, otherwise, the long-term outcomes will remain unsatisfactory [29].

## Treatment of obesity

Non-pharmacological treatment of obesity can be effective, but the long-term success rate is low and regaining lost weight is a major problem. Randomized studies have shown that a greater initial weight loss achieved with changes in lifestyle associated with other strategies (e.g. liquid formula diets or anorectic drugs) improves long-term weight maintenance, provided that it is followed by a 1–2 years of integrated weight maintenance programme consisting of lifestyle interventions involving dietary change, nutritional education, behaviour therapy and increased physical activity. Therefore, a greater initial weight loss as the first step with a pharmacological intervention may result in improved sustained weight maintenance [30].

Wing et al. demonstrated that the magnitude of weight loss at 1 year was strongly associated with improvements in blood pressure (BP), as well as fasting glucose, triglyceride, and HDL cholesterol levels but not in LDL cholesterol levels. Compared with weight-stable participants, those who lost 5 to <10% (7.25 ± 2.1 kg) of their body weight presented increased odds of achieving a 0.5%-point reduction in HbA1c, a 5-mmHg decrease in diastolic BP, a 5-mmHg decrease in systolic BP, a 5 mg/dL increase in HDL cholesterol, and a 40 mg/dL decrease in triglycerides. In those who lost 10–15% of their body weight, the odds of improvements were even greater [31].

Pharmacotherapy can be a useful choice for overweight/obese management. In general, long-term pharmacotherapy is recommended for use as an adjunctive treatment to lifestyle modification, enhancing its compliance [32]. Although anti-obesity drugs facilitate weight loss, the long-term therapy is frequently associated with a high dropout rate [33]. In the following subsections, we briefly review the new drugs for the treatment of obesity.

### Liraglutide 3.0 mg

The 3.0 mg dose of liraglutide was first approved in December 2014 for the treatment of obesity in the United States of America, being a higher dose than that already approved for second-line treatment of T2D [34].

**Fig. 2:**
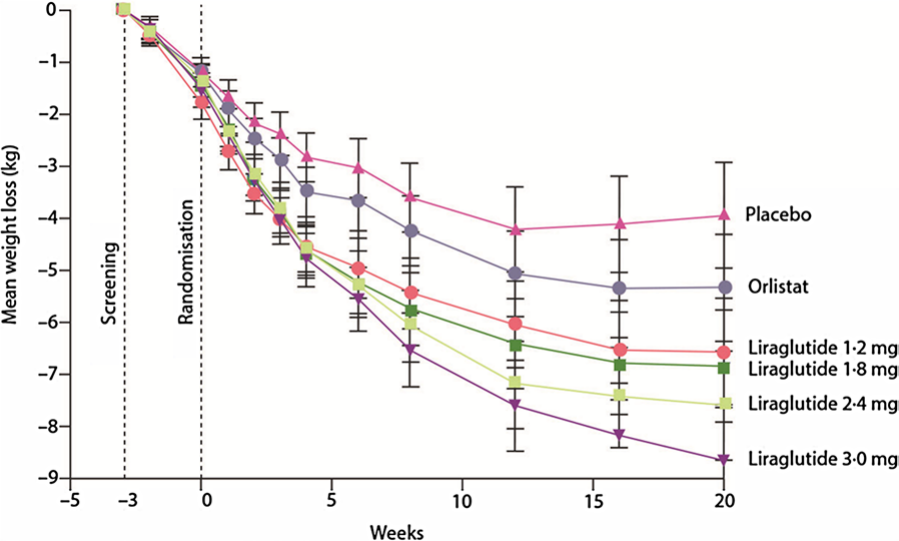
Change in body weight after treatment of obese individuals with four liraglutide doses (1.2, 1.8, 2.4, or 3.0 mg) or to placebo administered once a day subcutaneously, or orlistat (120 mg) three times a day orally. Data are mean (95% CI) for the ITT population with the last observation carried forward (LOCF) Adapted from Astrup et al. [35]

**Fig. 3:**
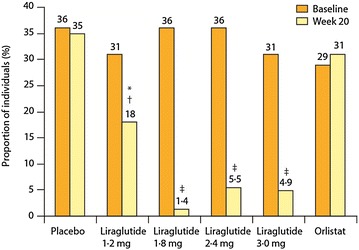
Proportion of individuals with prediabetes in the ITT population at randomisation and week 20. Individuals included are those with valid assessment at the start and the end of the 20-week trial period. *P = 0.007 vs placebo. ^†^P = 0.008 vs orlistat. ^‡^P ≤ 0.0001 vs placebo or orlistat Adapted from Astrup et al. [35]

Astrup et al. [35] reported a placebo-controlled 20-week trial, with orlistat as an active comparator. Patients (n = 564, BMI 30–40 kg/m^2^) were assigned to the following liraglutide doses: 1.2, 1.8, 2.4 and 3.0 mg (n = 90–95) or to placebo (n = 98) administered once a day (QD) subcutaneously, or to orlistat (120 mg; n = 95) 3 times a day orally. Additionally, participants had a 500 kcal per day energy-deficit diet and increased their physical activity. Weight change was analysed by intention to treat (ITT) and was the primary endpoint. An 84-week open-label extension followed. As shown in Fig. 2, liraglutide-induced a weight loss significantly higher than did placebo (P = 0.003 for liraglutide 1.2 mg and P < 0.0001 for liraglutide 1.8–3.0 mg) or orlistat (P = 0.003 for liraglutide 2.4 mg and P < 0.0001 for liraglutide 3.0 mg). Mean weight loss with liraglutide 1.2–3.0 mg was 4.8, 5.5, 6.3, and 7.2 kg compared with 2.8 kg with placebo and 4.1 kg with orlistat, and was 2.1 kg (95% CI 0.6–3.6) to 4.4 kg (2.9–6.0) greater than that with placebo. More individuals (76%, n = 70) lost more than 5% weight with liraglutide 3.0 mg that with placebo (30%, n = 29) or orlistat (44%, n = 42). Liraglutide reduced BP at all doses and reduced the prevalence of pre-diabetes (84–96% reduction) with 1.8–3.0 mg per day (Fig. 3).

In the phase 3 Scale Maintenance Study, obese/overweight individuals (≥18 years, BMI ≥30  or ≥27 kg/m^2^ with comorbidities) who lost greater than or equal to 5% of initial weight during a low-calorie diet in a 14 week-run-in were randomly assigned to liraglutide 3.0 mg per day or placebo for 56 weeks. Diet and exercise counselling were provided throughout the trial. Co-primary endpoints were the percentage of weight change from randomization, the proportion of participants that maintained the initial weight loss greater than or equal to 5%, and the proportion that lost ≥5% of randomization weight. In addition to the mean weight loss of 6.0 ± 0.9% achieved by the 422 participants during the run-in period, from randomization to week 56, weight decreased an additional mean of 6.2 ± 7.3% with liraglutide and 0.2 ± 7.0% with placebo (P < 0.0001). More participants in liraglutide group (81.4%) maintained the proposed run-in weight loss, compared with those in placebo group (48.9%) (P < 0.0001), and 50.5% vs. 21.8% of the participants lost ≥5% of randomization weight (P < 0.0001) [36].

**Fig. 4:**
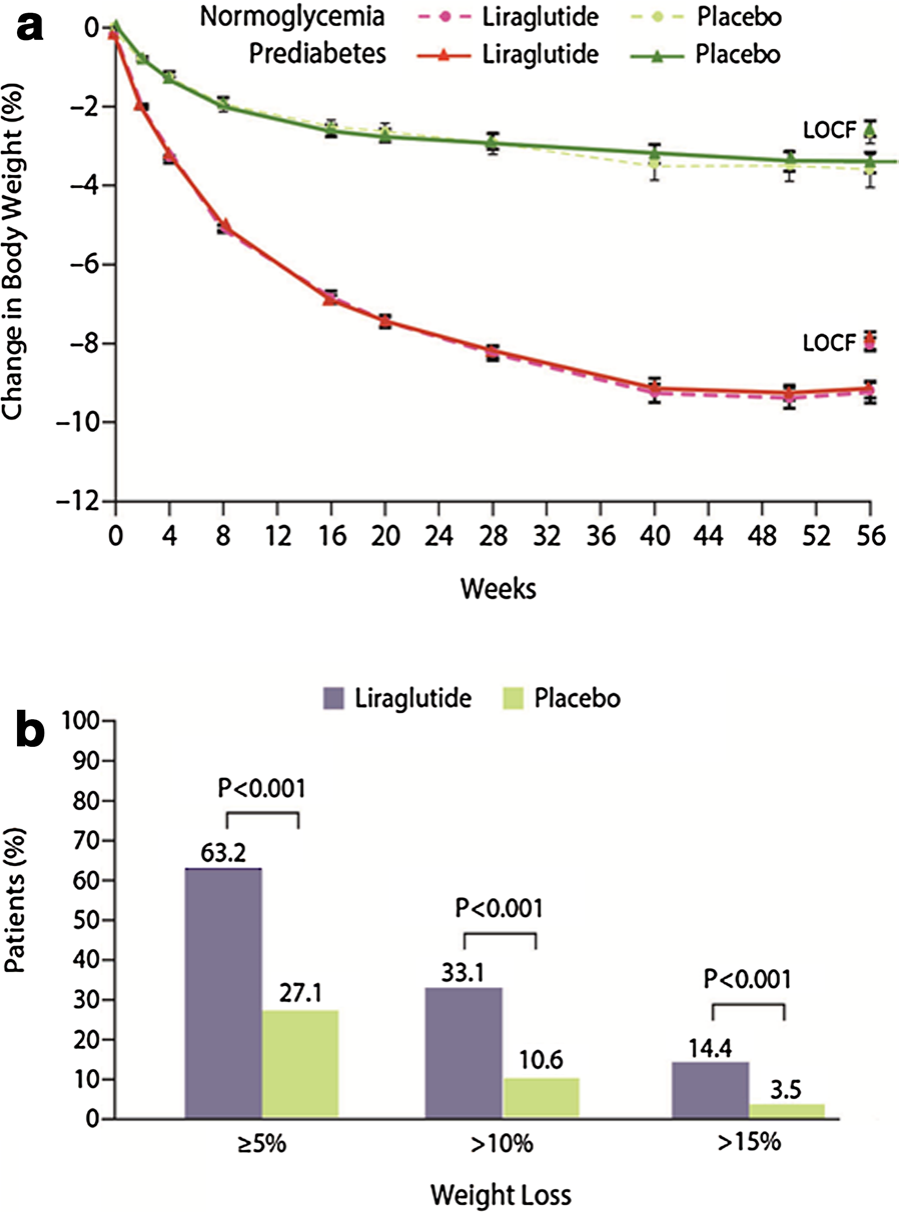
Mean body weight loss and categorical weight loss in the SCALE Obesity and Pre-diabetes study. **a** The mean body weight for patients in the full-analysis set who completed each scheduled visit, according to presence or absence of prediabetes at screening. *I bars* indicate standard error, and the *separate symbols above the curves* represent the 56-week weight change using last-observation-carried-forward (LOCF) imputation. Percentages of weight change in the liraglutide group were 8.0% with LOCF imputation and 9.2% for completers. In the placebo group, the changes were 2.6% with LOCF imputation and 3.5% for completers. The full-analysis set comprised patients who underwent randomization, were exposed to at least one treatment dose, and had at least one assessment after baseline (69 patients were excluded from the full-analysis set: 61 owing to lack of an assessment and 8 owing to no exposure). **b** The proportions of patients who lost at least 5%, more than 10%, and more than 15% of their baseline body weight. Data shown are the observed means for the full-analysis set (with LOCF). Findings from logistic-regression analysis showed an odds ratio of 4.8 (95% confidence interval [CI], 4.1 to 5.6) for at least 5% weight loss and an odds ratio of 4.3 (95% CI, 3.5 to 5.3) for more than 10% weight loss; the analysis of more than 15% weight loss was performed post hoc (odds ratio, 4.9 [95% CI, 3.5 to 6.7]) Adapted from Pi-Sunyer et al. [37]

In the SCALE Obesity and Pre-diabetes study, Pi-Sunyer et al. conducted a 56-week, double-blind trial including 3.731 patients without T2D and a BMI ≥30  or ≥27 kg/m^2^ if they had dyslipidemia or hypertension [37]. Patients were allocated in a 2:1 ratio to receive once-daily subcutaneous injections of liraglutide at a dose of 3.0 mg (2.487 patients) or placebo (1.244 patients), and were advised on lifestyle changes. The co-primary endpoints were the variation in body weight and the proportions of patients losing at least 5% and more than 10% of their initial body weight. Patients in the liraglutide group lost a mean of 8.4 ± 7.3 kg of body weight, and those in the placebo group lost a mean of 2.8 ± 6.5 kg (a difference of −5.6 kg; P < 0.001). A total of 63.2% of the patients in the liraglutide group vs. 27.1% in the placebo group lost at least 5% of their body weight (P < 0.001), and 33.1 and 10.6%, respectively, lost more than 10% of their body weight (P < 0.001, Fig. 4). There was a greater reduction in HbA1c, fasting glucose, and fasting insulin levels, as well as in plasma glucose levels during an oral glucose-tolerance test (OGTT) in the liraglutide rather than in the placebo group and higher insulin and C-peptide levels relative to placebo during the OGTT. These effects were more noticeable in prediabetic patients than in normoglycaemic ones. At week 56, the prevalence of prediabetes was significantly lower in the liraglutide than in the placebo group and also T2D developed in more patients in the placebo than in the liraglutide group during the course of treatment [37]. These effects could probably be attributed to the combination of weight loss and improved glycemic control with liraglutide.

Systolic and diastolic BP decreased more in the liraglutide group than in the placebo group by week 56 and levels of fasting lipids, high-sensitivity C-reactive protein, plasminogen activator inhibitor-1, and adiponectin exhibited more improvement in the liraglutide group than in the placebo group [37]. Treatment with liraglutide was linked with improvements in health-related quality of life, notably physical function, as compared with placebo [37].

Regarding adverse events, gastrointestinal events were the most common side effects and were reported more frequently with liraglutide than placebo. Nausea and vomiting occurred more often in individuals on liraglutide than in those on placebo, mainly in the 4–8 weeks after initiation of treatment, but were mainly transient and rarely led to discontinuation of treatment [37].

Rare adverse effects consisted of pancreatitis (most gallstone-related pancreatitis), cholelithiasis and cholecystitis. The positive predictive value of isolated lipase or amylase enzyme elevations for diagnosing pancreatitis was very low (<1% for a lipase value ≥3 times the upper limit of normal [ULN] range and among patients who had pancreatitis no one had amylase values ≥3 times the upper limit of the normal range [37]. Lipase may be elevated in asymptomatic obese (5%) and T2D (20%) patients already before treatment. In asymptomatic T2D patients, lipase levels can be greater than 2 times the ULN in 4.5% and 3 times the ULN in 2.1% of them [38]. In a report from the LEADER (Liraglutide Effect and Action in Diabetes: Evaluation of Cardiovascular Outcome Results) trial, nearly 25% of T2D patients had elevated lipase or amylase levels without symptoms of acute pancreatitis, before randomization to liraglutide or placebo [39]. Acute pancreatitis occurred in 18 patients in the liraglutide group and in 23 in the placebo group 54 months after randomization [40].

Because of an increased incidence of thyroid C-cell tumours in rodents, the FDA states that liraglutide is contraindicated in those with a personal or family history of medullary thyroid cancer or multiple endocrine neoplasias (MEN) syndrome type 2. In the SCALE trial and also in the LEADER trial there were no cases of medullary thyroid carcinoma or C-cell hyperplasia and liraglutide treatment did not increase serum calcitonin concentrations [37, 40]. A meta-analysis including 25 studies aimed to evaluate the risk of serious adverse events associated with liraglutide and exenatide in patients with T2D. Liraglutide did not increase the risk of acute pancreatitis (0.97 [95% CI 0.21–4.39]), cancer (1.35 [95% CI 0.70, 2.59]), or thyroid cancer (1.54 [95% CI 0.40–6.02]) [41]. Pancreatitis seems to be associated with the onset of weight loss-induced gallstone formation [42].

The long-term cardiovascular outcome safety of liraglutide was formally evaluated in the LEADER trial, which randomized 9.340 T2D patients for treatment with liraglutide 1.8 mg or placebo for a period of up to 5 years. Liraglutide significantly reduced the risk of major adverse cardiovascular events, including death from cardiovascular causes, non-fatal myocardial infarction, or non-fatal stroke [40].

#### No weight-loss related effects of liraglutide

It is suggested that liraglutide exerts cardioprotective effects. More detailed explanations regarding the cardiovascular effects of GLP-1R agonists can be found in reviews authored by Saraiva and Sposito [43] and Drucker [44]. Moreover, this class of drugs has been showing protective effects in several different tissues, including brain [45]. The Imperial College of Science, in London, is conducting phase 2 trials for the treatment of patients with mild Alzheimer’s disease (ClinicalTrials.gov Identifier: NCT01469351).

### Lorcaserin

Lorcaserin is a selective serotonin 2C receptor [5-hydroxytryptamine 2C (5-HT2C) receptor] agonist that acts on the hypothalamus (Fig. 1) to increase satiety [46]. The FDA approved lorcaserin in 2012 at a dose of 10 mg twice daily (BID) for long-term treatment of obesity based on the results of three key randomized clinical trials. The BLOOM (Behavioral Modification and Lorcaserin for Overweight and Obesity Management) study was a double-blind clinical trial, with 3.182 obese or overweight adults to receive lorcaserin at a dose of 10 mg, or placebo, BID for 52 weeks, besides diet and exercise counselling. At week 52, patients in the placebo group continued to receive placebo but patients in the lorcaserin group were randomly reassigned to receive either placebo or lorcaserin. At 1 year, 47.5% of patients in the lorcaserin group and 20.3% in the placebo group had lost 5% or more of their body weight (P < 0.001), corresponding to an average loss of 5.8 ± 0.2 kg with lorcaserin and 2.2 ± 0.1 kg with placebo during year 1 (P < 0.001). Among the patients who received lorcaserin during year 1 and who had lost 5% or more of their baseline weight at 1 year, the loss was maintained in more patients who continued to receive lorcaserin during year 2 (67.9%) than in patients who received placebo during year 2 (50.3%, P < 0.001) [47].

The BLOSSOM (Behavioral Modification and Lorcaserin Second Study for Obesity Management) was a 1-year randomized placebo-controlled, double-blind, parallel arm trial that included 4008 obese and overweight patients. Patients were randomly assigned in a 2:1:2 ratio to receive lorcaserin 10 mg BID, lorcaserin 10 mg QD, or placebo. All patients received diet and exercise counseling. Significantly more patients treated with lorcaserin 10 mg BID and QD lost at least 5% of baseline body weight (47.2 and 40.2%, respectively) as compared with placebo (25.0%, P < 0.001 vs. lorcaserin BID). Least squares mean (95% confidence interval) weight loss with lorcaserin BID and QD was 5.8% (5.5–6.2%) and 4.7% (4.3–5.2%), respectively, compared with 2.8% (2.5–3.2%) with placebo (P < 0.001 vs. lorcaserin BID; least squares mean difference, 3.0%). Weight loss of at least 10% was achieved by 22.6 and 17.4% of patients receiving lorcaserin 10 mg BID and QD, respectively, and 9.7% of patients in the placebo group (P < 0.001 vs. lorcaserin BID) [48].

**Fig. 5:**
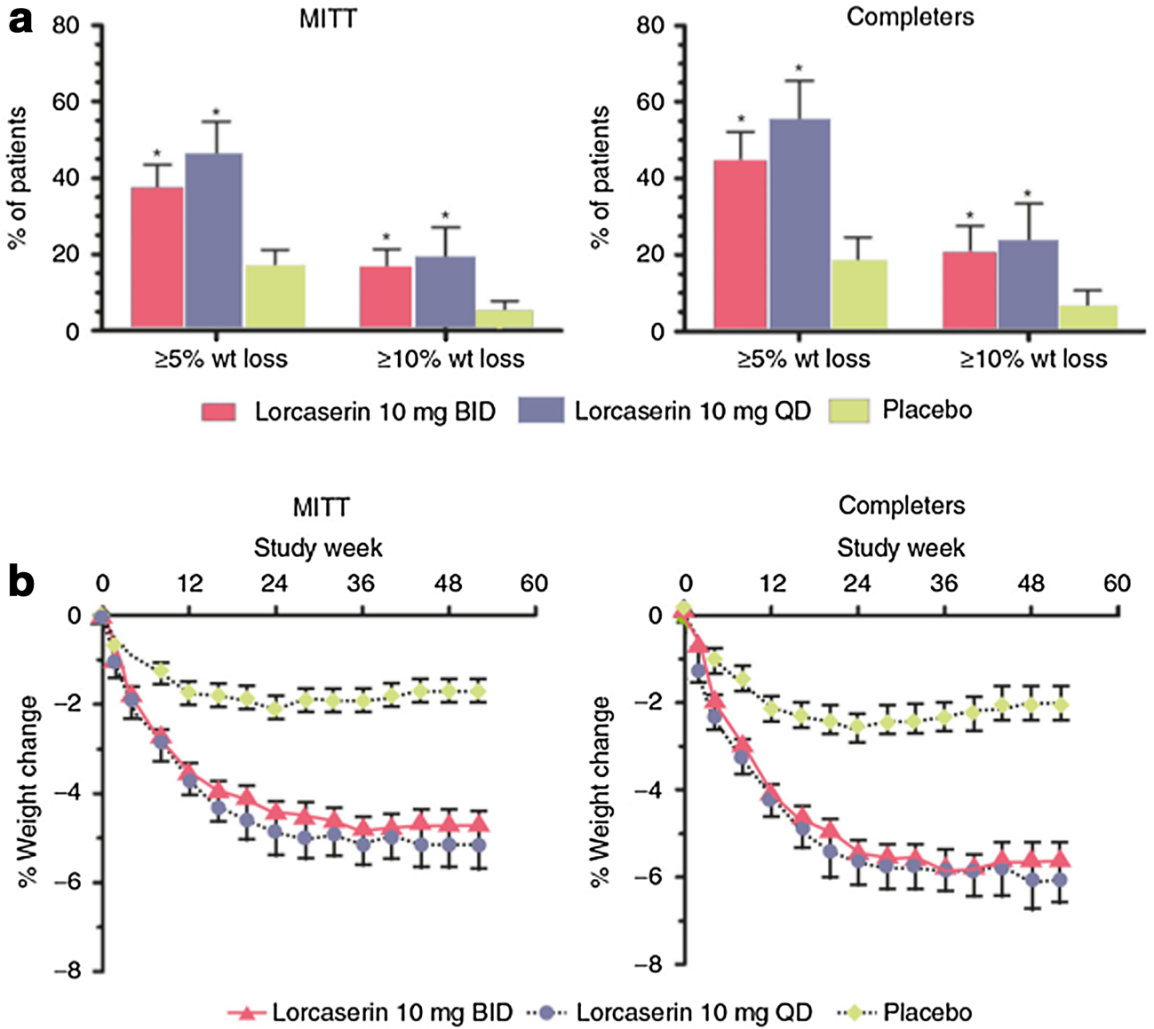
Categorical body weight change and mean body weight loss in the BLOOM-DM trial. **a** Proportion of patients who lost ≥5 or ≥10% of body weight from baseline to week 52 using the modified intent to treat (MITT) population (*left panel*) or the completers population (*right panel*). Lorcaserin 10 mg BID *red bars*; lorcaserin 10 mg QD *blue bars*; placebo *green bars*. Values are proportion ± 95% confidence interval. *P < 0.001 as compared to placebo. **b** Percent change in body weight from baseline to each study visit, using the MITT population (*left panel*) or the completers population (*right panel*). Lorcaserin 10 mg BID *red triangles with solid line*; lorcaserin 10 mg QD *blue circles with dashed line*; placebo *green diamonds with dashed line*. Values are mean ± SEM. BID, twice daily; QD, once daily Adapted from O’Neil et al. [49]

The BLOOM-DM (Behavioral Modification and Lorcaserin for Obesity and Overweight Management in Diabetes Mellitus) study evaluated efficacy and safety of lorcaserin for weight loss in T2D patients. This 1-year, randomized, placebo-controlled trial enrolled 604 patients 1:1:1 to placebo, lorcaserin 10 mg QD or lorcaserin 10 mg BID and received diet and exercise counselling. Lorcaserin significantly increased the proportion of patients achieving ≥5% body weight loss from baseline to week 52 relative to placebo (Fig. 5). Using modified ITT (mITT) with last-observation-carried-forward (LOCF) imputation, 37.5% of patients on lorcaserin BID, 44.7% on lorcaserin QD, and 16.1% of patients on placebo lost at least 5% (*P* < 0.001), while 16.3, 18.1, and 4.4%, respectively, lost at least 10% of baseline body weight (*P* < 0.001). The weight reduction remained significantly greater in the lorcaserin groups than in the placebo group throughout the study (Fig. 5). Similar results were obtained when data from the subgroup of patients who completed the 52-week trial were analyzed (the completer population) [49].

The analysis of pooled data from the BLOOM and the BLOSSOM studies showed that at week 52, more than twice as many lorcaserin-treated patients achieved a weight loss of ≥5% compared with placebo. A significantly greater proportion of lorcaserin-treated patients achieved a weight loss of ≥10% (lorcaserin, 22.4%; placebo, 8.7%). There were also clinically relevant improvements in cardiometabolic parameters, with significant improvements in lipid parameters, glycemic indicators, quality-of-life measures, and vital signs in the lorcaserin group compared with placebo [50].

At first, some possibly serious safety concerns about lorcaserin have been pointed out, mostly a numerical disproportion in the incidence of valvulopathy. That was worrisome, provided that the weight-loss drugs fenfluramine and dexfenfluramine, activating 5-HT2B receptors on interstitial heart cells, were removed from the market in 1997 owing to an association with valvulopathy. Integrated data analysis from 3 phase 3 trials with 5249 obese and overweight patients treated with 10 mg lorcaserin BID or placebo was 52 weeks showed that the relative risk of valvulopathy in lorcaserin-treated participants as compared with the placebo group, was 1.16 (95% CI, 0.81–1.67). These results may be partially influenced by greater weight loss in the lorcaserin group than in the placebo group. Even though not statistically significant, the 16% increase in the risk gave cause for some concern [51]. Nevertheless, in vitro receptor assays revealed that lorcaserin has a very much greater selectivity for the 5-HT2C than for the 5HT2B receptor and would not be expected to increase the risk of valvulopathy in humans [52].

The most common adverse events of lorcaserin are headache, nausea, dizziness, fatigue, dry mouth, and constipation, but otherwise, it is well tolerated [48, 50]. In patients on concomitant use of selective serotonin re-uptake inhibitors (SSRIs), lorcaserin can theoretically increase the risk of serotonin syndrome [53, 54].

The value of lorcaserin seems to be on its safety and tolerability, but not on the magnitude of the weight loss. Its efficacy was marginal according to the FDA efficacy standard for weight loss medications (the first benchmark, treated patients met a mean weight loss at least 5% greater than that of patients receiving placebo was not achieved in any study, but it met the second benchmark, with over 35% of subjects losing 5% or more of their baseline weight) [54].

### Phentermine/topiramate combination

The combination of phentermine (PHEN) and controlled-release topiramate (TPM CR) is indicated for long-term treatment of obesity. Phentermine acts to reduce appetite through increasing norepinephrine in the hypothalamus and TPM may reduce appetite through its effect on GABA receptors (Fig. 1) [55]. The combination comprises lower doses of PHEN than the ones used as single agent (3.75 mg in the starting dose, 7.5 mg in the recommended dose and 15 mg in the full dose). The doses of TPM CR (23 mg in the starting dose, 46 mg in the recommended dose and 92 mg in the full dose) are also lower than the ones used for migraine prophylaxis or seizures control [55].

**Fig. 6:**
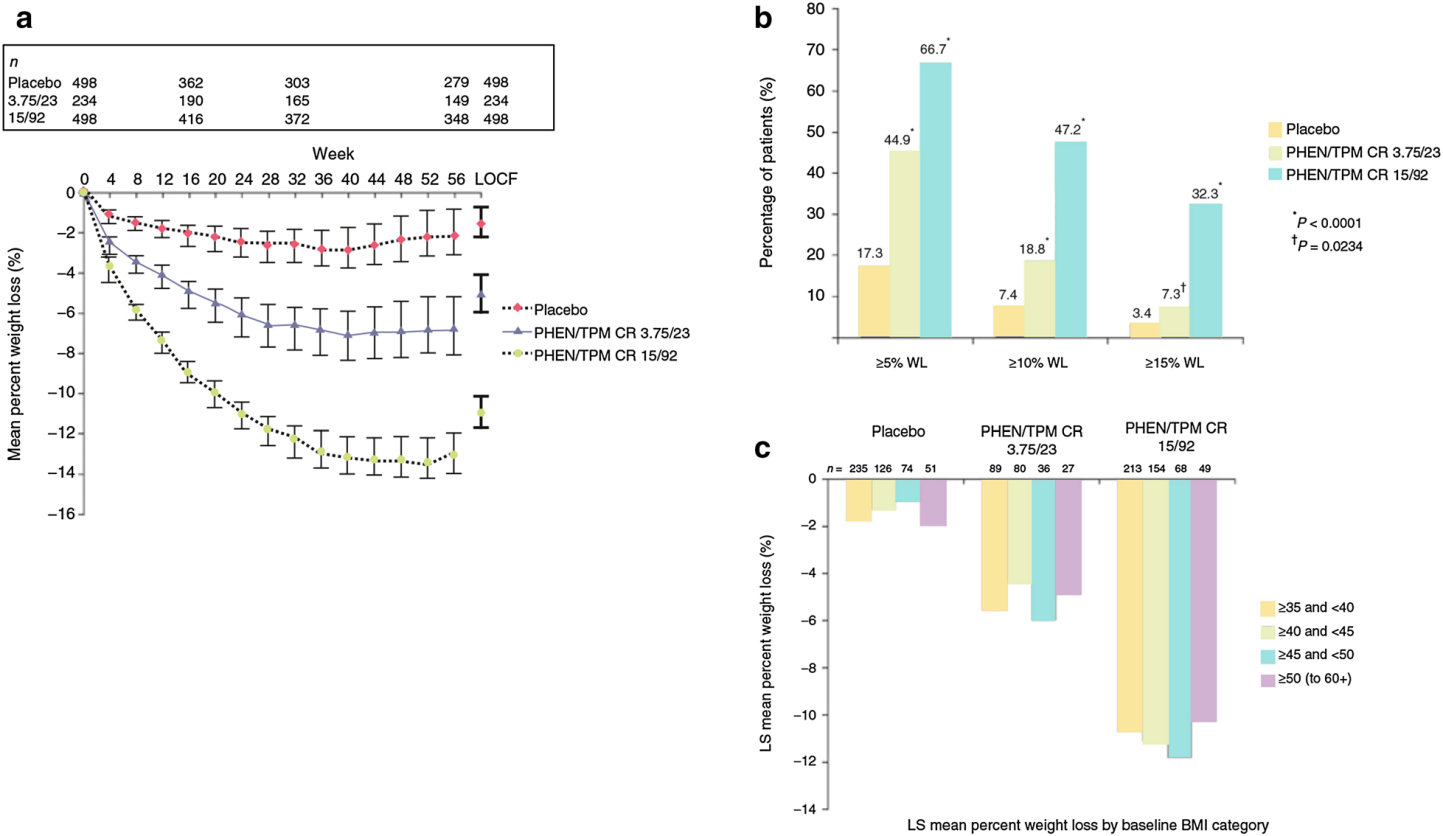
Mean weight loss, categorical weight loss, and percent weight loss by baseline BMI category in the EQUIP trial. Efficacy results are shown with analysis A (prespecified ITT/LOCF). **a** Mean percent weight loss; **b** Patients achieving ≥5, ≥10, and ≥15% WL; **c** LS mean percent weight loss by baseline BMI category. Error bars represent 95% confidence interval. ITT, intent-to-treat; LOCF, last observation carried forward; LS, least-squares; PHEN/TPM CR, controlled-release phentermine/topiramate Adapted from Allison et al. [56]

In the EQUIP trial, obese subjects were randomized to placebo, PHEN/TPM CR 3.75/23 mg, or PHEN/TPM CR 15/92 mg, added to a reduced-energy diet. Regardless of analysis used, patients in the 15/92 group lost significantly (*P* < 0.0001 for all comparisons) more weight than patients in the 3.75/23 group who in turn lost significantly more weight than patients receiving placebo (Fig. 6). Patients in the placebo, 3.75/23, and 15/92 groups lost 1.6, 5.1, and 10.9% of baseline body weight, respectively, at 56 weeks. Proportions of patients achieving 5% weight loss were 17.3% of placebo patients, 44.9% of 3.75/23 patients, and 66.7% of 15/92 patients [56].

The CONQUER trial included overweight or obese patients with a BMI of 27–45 kg/m^2^ and at least two metabolic syndrome comorbidities. Of 2.487 patients, 994 were assigned to placebo, 498 to PHEN 7.5 mg plus TPM 46.0 mg, and 995 to PHEN 15.0 mg plus TPM 92.0 mg. The combination PHEN/TPM CR promoted weight losses approaching 10%. The extension for the second year of observation was called SEQUEL [57], with patients keeping their treatment regimen. At the end of 2 years, patients completing the trial taking 7.5 mg/46 mg maintained a weight loss of 9.3% below baseline and those on the higher doses maintained a 10.5% weight loss from baseline [58].

In the EQUIP, CONQUER and SEQUEL studies, improvements in risk factors were related to the amount of weight loss, with the greater benefit being observed with greater weight loss. The most commonly observed side effects in these clinical trials were paresthesias, cognitive impairment, dizziness, dysgeusia, insomnia, constipation, metabolic acidosis and dry mouth. Glaucoma is a rare side effect of TPM, and the drug is contraindicated when this condition is present [53]. During the 2-year period in the CONQUER/SEQUEL study, the incidence of reported psychiatric anxiety-related adverse events was dose-related: 3.1, 6.5, and 9.5% for placebo, 7.5/46, and 15/92 arms, respectively. Potentially serious safety concerns regarding PHEN/TPM CR included teratogenicity (orofacial cleft) [59] and elevations in resting heart rate [58]. Subsequently, the approval of PHEN/TPM CR demanded a risk evaluation and mitigation strategy (REMS). This included a medication guide, a patient brochure, a formal training program for prescribers (both the brochure and the program inform about the teratogenic risk and stress the need for women with reproductive potential to use effective forms of contraception and also recommend pregnancy testing before initiating treatment and monthly during the follow-up) and authorized only especially accredited drugstores to dispense PHEN/TPM CR [54]. The drug’s labeling advises frequent heart rate monitoring and not to use in patients with recent or unstable cardiac or cerebrovascular disease since the combination has not been investigated in this particular patient cohort. It is worth to emphasize that the effect on weight loss observed with this combination is the result of the administration of two active pharmaceutical ingredients.

### Naltrexone/bupropion sustained-release (SR)

Naltrexone is an opioid receptor antagonist with minimal effect on weight loss on its own. Bupropion reduces food intake by acting on adrenergic and dopaminergic receptors in the hypothalamus. Even so, the association of both has shown a synergistic effect. Bupropion stimulates the cleavage of POMC and at the same time that increases the agonism of melanocortin-4 receptor by releasing α-MSH. Simultaneously, other cleavage product of POMC, ß-endorphin induces a negative autologous feedback loop in the POMC neuron itself when it binds at the μ opioid receptor, reducing neuronal activity. The ß-endorphin opioid-mediated effect is blocked by the opioid receptor antagonist naltrexone, which amplifies the effect of α-MSH to reduce food intake (Fig. 1) [60].

**Fig. 7:**
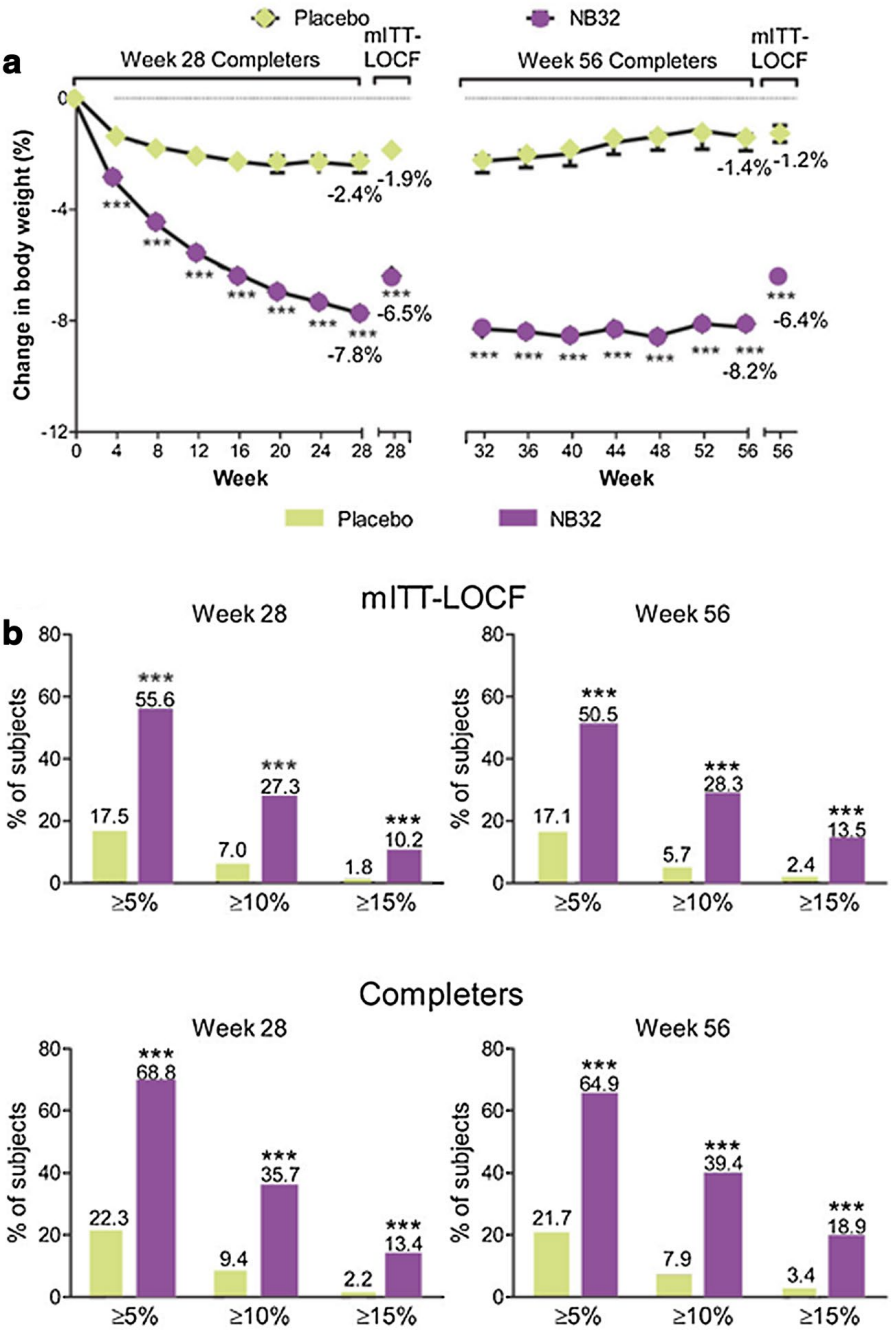
Mean weight loss and categorical weight loss in the COR-II trial. **a** Percent weight loss (observed; LS mean ± SE) by visit in the week 28 and 56 completers (NB32 data are weighted for weeks 32–56), and percent weight loss for the week 28 and 56 mITT-LOCF subjects. **b** Categorical weight loss in week 28 and 56 mITT-LOCF and completers. ***P < 0.001 for NB32 vs. placebo. mITT analysis: prespecified modified intent-to-treat population composed of all randomized participants with a baseline weight and ≥1 post-baseline weight on study drug (+1 day post-last dose); LOCF: missing data were imputed by carrying forward the last observation on study drug; completers: participants who completed 28 or 56 weeks of treatment Adapted from Apovian et al. [62]

The four 56-week placebo-controlled, randomized COR (Contrave Obesity Research) trials [COR-I, COR-II, COR–Behaviour Modification (COR-BMOD), and COR-Diabetes (COR-D)] assessed the efficacy of naltrexone/bupropion SR [61–64]. In overweight and obese patients without T2D, the placebo-subtracted weight loss ranged from 4.2% in the COR-BMOD to 4.8% in the COR-I trial using the highest dose (32 mg/360 mg) of naltrexone/bupropion SR. In patients with T2D, as always, the placebo-subtracted weight loss was less effective [61, 63]. The COR-II trial was a phase 3 study that involved 1.496 patients with overweight or obesity with controlled hypertension and/or dyslipidemia. Patients were randomized in a 2:1 fashion to 32 mg/day naltrexone SR plus 360 mg/day bupropion SR (NB32) or placebo for up to 56 weeks. In the mITT-LOCF population, weight loss was significantly greater for NB32 versus placebo at week 28 (6.5% vs. 1.9%; P < 0.001). Weight loss was maintained with continued double-blind treatment in the NB32 group through week 56 (6.4% vs. 1.2; P < 0.001). NB32 was associated with a significantly larger proportion of participants achieving 5, 10, and 15% weight loss in the mITT-LOCF population versus placebo at weeks 28 and 56 (Fig. 7). Treatment also was linked to significant improvements in control of eating, weight-related quality of life, and cardiovascular risk factors.

The most common adverse effects of this combination were nausea, constipation, headache, vomiting, and dizziness. Bupropion has the potential of increasing the risks for suicidality and for neuropsychiatric symptoms. Nausea and vomiting were the adverse effects that led to a dropout rate of almost 50% in the clinical studies. To improve tolerability, the tablets are presented in a combination of 90 mg bupropion SR and 8 mg naltrexone SR, allowing titration with progressive increase of 1 tablet every week until a final total dose of 2 tablets BID [64].

The weight loss achieved at 1 year with the naltrexone/bupropion combination was intermediate between PHEN/TPM CR and lorcaserin and associated with improvement in risk factors. However, the decline in blood pressure is not as great as one would expect from the weight loss in the phase 3 trials of naltrexone/bupropion [53].

Table 1 summarizes contraindications, drug interactions, common adverse effects and stopping rules of liraglutide, lorcaserin, phentermine/topiramate and naltrexone/bupropion [65–68].

**Table 1:** Main contraindications, drug interactions, common adverse effects and stopping rules of liraglutide, lorcaserin, phentermine/topiramate and naltrexone/bupropion

Drug name	Contraindications	Drug interactions	Common adverse effects	Stopping rule
Liraglutide	It is unknown whether liraglutide causes thyroid C-cell tumors, including medullary thyroid carcinoma (MTC), in humans, as the human relevance of liraglutide-induced rodent thyroid C-cell tumors has not been determined. Also it is contraindicated in patients with a personal or family history of MTC or in patients with Multiple endocrine neoplasia syndrome type 2. Pregnant women or women who are nursing	Concurrent oral drug requiring rapid onset (it can delay gastric emptying)	Nausea, hypoglycemia [serious hypoglycemia can only occur when liraglutide is used with an insulin secretagogue (e.g. a sulfonylurea)], diarrhea, constipation, vomiting, headache, decreased appetite, dyspepsia, fatigue, dizziness, abdominal pain, and increased lipase	Stop if <4% weight loss at 16 weeks
Lorcaserin	Pregnant women, or women who are nursing	Based on the mechanism of action of lorcaserin and the theoretical potential for serotonin syndrome, use with extreme caution in combination with other drugs that may affect the serotonergic neurotransmitter systems, including, but not limited to, triptans, monoamine oxidase inhibitors (MAOIs, including linezolid, an antibiotic which is a reversible nonselective MAOI), selective serotonin reuptake inhibitors (SSRIs), selective serotonin-norepinephrine reuptake inhibitors (SNRIs), dextromethorphan, tricyclic antidepressants (TCAs), bupropion, lithium, tramadol, tryptophan, and St. John’s Wort	In patients without diabetes: headache (17%), dizziness (9%), fatigue (7%), nausea (8%), dry mouth (5%), and constipation (6%). In patients with diabetes: hypoglycemia (29%), headache (15%), back pain (12%), cough (8%), and fatigue (7%)	Stop if <5% loss at 12 weeks
Phentermine/topiramate CR	Pregnancy (teratogenic risk), glaucoma, hyperthyroidism, during or within 14 days of taking MAOIs, known hypersensitivity or idiosyncrasy to sympathomimetic amines. Should not be used by nursing mothers	Oral contraceptives: altered exposure may cause irregular bleeding but not increased risk of pregnancy; CNS depressants including alcohol: potentiate CNS depressant effects. Non-potassium sparing diuretics: may potentiate hypokalemia. Antiepileptic drugs: concomitant administration of phenytoin or carbamazepine with topiramate in patients with epilepsy, decreased plasma concentrations of topiramate by 48 and 40%, respectively, when compared to topiramate given alone. Concomitant administration of valproic acid and topiramate has been associated with hyperammonemia (with and without encephalopathy) and hypothermia (with and without hyperammonemia)	Paresthesias, dizziness, taste alterations, insomnia, constipation, dry mouth, elevation in heart rate, memory and cognitive changes, secondary acute angle closure glaucoma, suicidal behavior and ideation, fetal toxicity, mood and sleep changes, metabolic acidosis	If 3% weight loss is not achieved with 7.5 mg/46 mg dose after 12 weeks, stop or increase to 11.25 mg/69 mg for 14 days, then 15 mg/92 mg; Stop if <5% loss at 12 weeks on top dose
Naltrexone/bupropion	Uncontrolled hypertension; seizure disorders, anorexia nervosa or bulimia, or undergoing abrupt discontinuation of alcohol, benzodiazepines, barbiturates, and antiepileptic drugs; use of other bupropion-containing products; chronic opioid use; during or within 14 days of taking MAOIs; pregnant women or women who are nursing	MAOIs: Increased risk of hypertensive reactions can occur when used concomitantly. Bupropion inhibits CYP2D6 and can increase concentrations of: antidepressants, (e.g., selective serotonin reuptake inhibitors and many tricyclics), antipsychotics (e.g., haloperidol, risperidone and thioridazine), beta-blockers (e.g., metoprolol) and Type 1C antiarrhythmics (e.g., propafenone and flecainide). Concomitant treatment with CYP2B6 Inhibitors (e.g., ticlopidine or clopidogrel) can increase bupropion exposure. Do not exceed one tablet twice daily when taken with CYP2B6 inhibitors. CYP2B6 Inducers (e.g., ritonavir, lopinavir, efavirenz, carbamazepine, phenobarbital, and phenytoin) may reduce efficacy by reducing bupropion exposure, avoid concomitant use. Dopaminergic drugs (levodopa and amantadine): CNS toxicity can occur when used concomitantly with natrexone/buprobion combination. Drug-laboratory test interactions: natrexone/buprobion can cause false-positive urine test results for amphetamines	Constipation, headache, nausea, vomiting, dizziness, insomnia, dry mouth and diarrhea	Stop if <5% loss at 12 weeks

## Conclusion

Obesity is a chronic, progressive, multifactorial disease determined by genetic and environmental factors; its complex pathophysiology and the intrinsic difficulties associated with maintenance of lifestyle modifications contribute to the unsatisfactory long-term outcomes observed in the obesity treatment. Just as with other chronic diseases, pharmacological interventions are useful to maximize non-pharmacological approaches in the long-term management of this condition.
